# Supplementary material for: Inducing cancer indolence by targeting mitochondrial Complex I is potentiated by blocking macrophage-mediated adaptive responses
Source: Nat Commun. 2019 Feb 22;10:903. doi: 10.1038/s41467-019-08839-1 (PMC6385215; doi:10.1038/s41467-019-08839-1)
Supplement: Supplementary file 1 — Supplementary Information [file 41467_2019_8839_MOESM1_ESM.pdf]

Supplementary information

Inducing cancer indolence by targeting mitochondrial Complex I is potentiated by blocking macrophage-mediated adaptive responses

Kurelac et al.

Supplementary Figures

a

Microsatellite	Query	DMSZ Result	Query	DMSZ Result
	143B$^{+/+}$	143.98.2 (CRL-11226)	HCT$^{+/+}$	HCT116 (CCL-247)
D5S818	13	13,13	8,10	10,11
D13S317	12	12,12	10,12	10,12
D7S820	11,12	11,12	11,12	11,12
D16S539	10,13	10,13	12,13	11,13
VWA	18	18,18	15,16,17	17,22
TH01	6	6,6	8,9	8,9
AM	X	X,X	X	X,Y
TPOX	11	11,11	8	8,9
CSF1PO	12,13	12,12	7,10	7,10

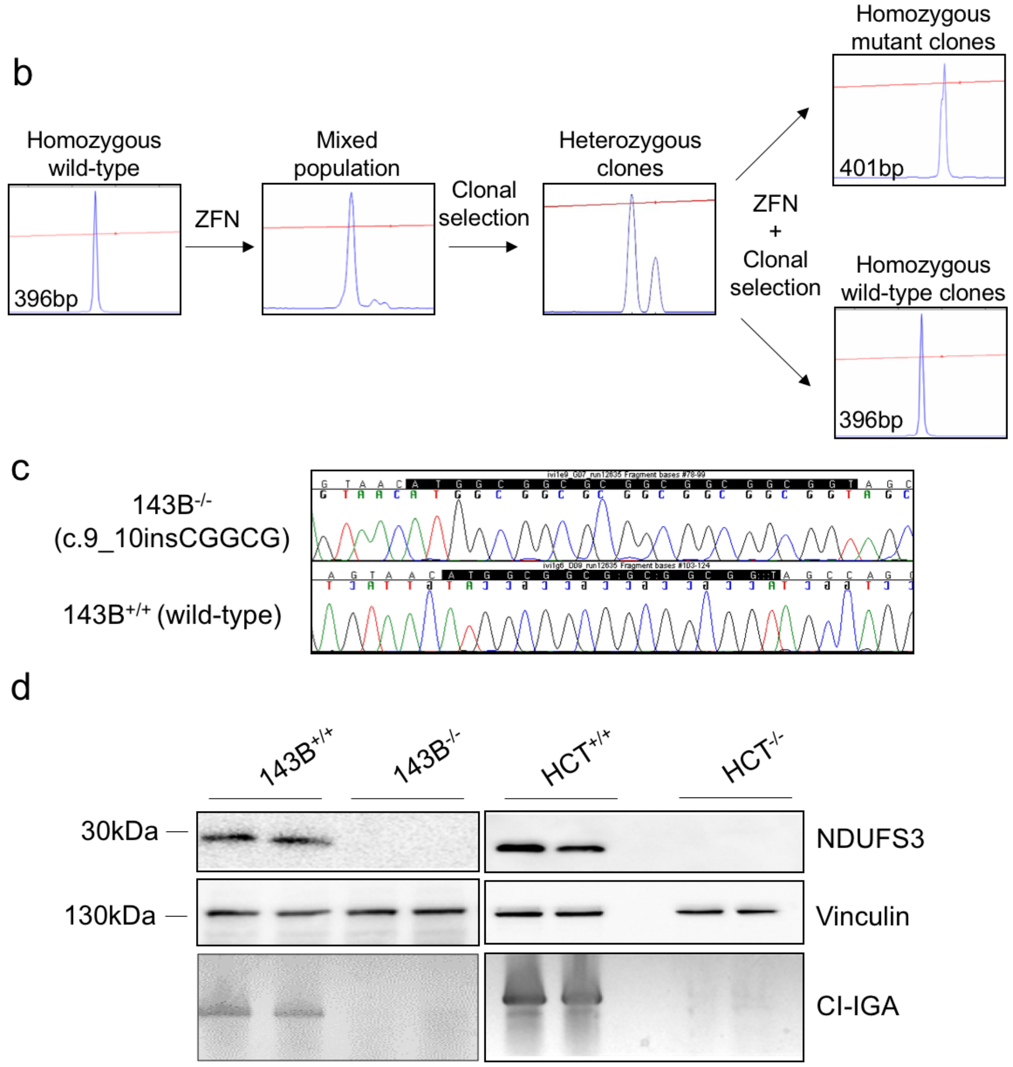

Supplementary Figure 1. Generation and characterization of cell models used in this study. (a) Evaluation of the sequence tandem repeats (microsatellites) defining the origin of 143B and HCT116 (HCT) cell lines. The results of the best match provided by the Online STR Analysis software (www.dmsz.de) are reported in the blue columns. **(b)** Fluorescent-PCR data in a scheme representing the generation of zinc-finger endonuclease (ZFN)-mediated *NDUFS3* knock-out cells and respective revertant wild-type controls. bp: base pairs. **(c)** Sanger sequencing-derived electropherograms showing the frameshift c.9_10insCGGCG mutation causing *NDUFS3* functional knock-out. **(d)** NDUFS3 western blot and CI In-Gel Activity (CI-IGA) of control (+/+) and *NDFUS3* knock-out (-/-) cells. Vinculin is used as loading control.

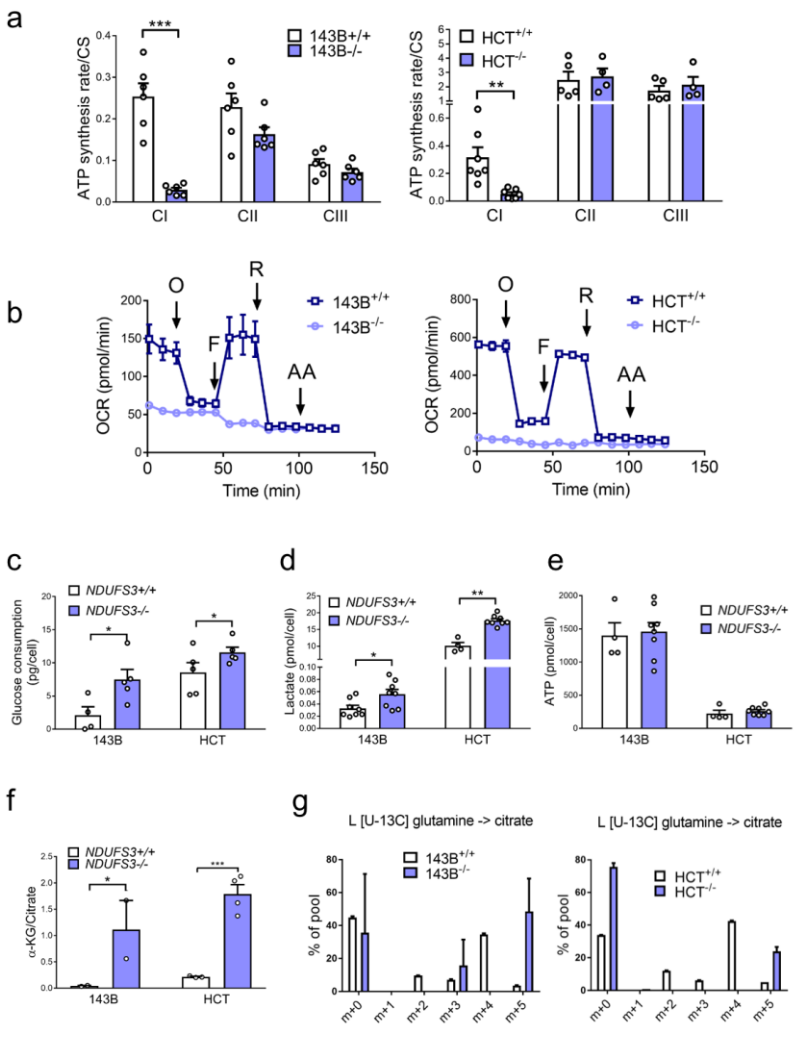

Supplementary Figure 2. Biochemical characterization of NDUFS3 KO cells. (a) Rate of mitochondrial ATP synthesis in digitonin-permeabilized 143B (data were log transformed, n=6, df=10, t=9.3) and HCT (data were log transformed, n=7, df=12, t=5.4) cells driven by pyruvate/malate (CI), succinate (CII) or DBH_2 (CIII). Data (mean+s.e.m.) are normalized on citrate synthase (CS) activity and protein content. **(b)** Oxygen consumption rate (OCR) profile of 143B (n=3) and HCT (n=1) cells determined upon injection of 1 µM oligomycin (O), 1 µM rotenone (R) and 1 µM antimycin A (A) in specific Seahorse medium. FCCP (F) concentration was determined by titration and used 0.5 µM for CI-competent cells and 0.1 µM for CI-deficient cells. Data (mean±s.e.m.) are normalized on SRB absorbance. **(c)** Glucose consumption determined in 143B (n=5, df=8, t=2.6) and HCT cells (n=5, df=8, t=2.5) cultured in basal conditions for 24 hours. Data (mean+s.e.m.) are normalized on cell number. **(d)** L-lactate production in 143B (n≥6, df=12, t=2.3) and HCT cells (n≥4, df=10, t=7.5) cultured in basal conditions for 24 hours. Data (mean+s.e.m.) are normalized on cell number. **(e)** Total ATP amount measured in 143B (n≥4) and HCT cells (n≥4) cultured in basal conditions. Data (mean+s.e.m.) are normalized on cell number. **(f)** α-KG/citrate ratio in 143B (data were log transformed, n=2, df=2, t=5.4) and HCT (data were log transformed, n≥3, df=5, t=16.7) cell lines cultured in basal conditions. Data are mean+s.e.m. **(g)** Isotope-labelled carbon (^{13}C) incorporation into citrate upon 5 hours incubation with 13C-glutamine (2 mM), in 143B (n=2) and HCT (n=2) cells. Data are expressed as percentage of citrate total levels and are mean+s.e.m. **(h)** Cell viability under basal conditions determined by SRB assay. Data are mean±s.e.m. [n=3, df=4, t(72h)=6.2]. In each panel, statistical significance is specified with asterisks (*p < 0.05, **p < 0.01, ***p < 0.001).

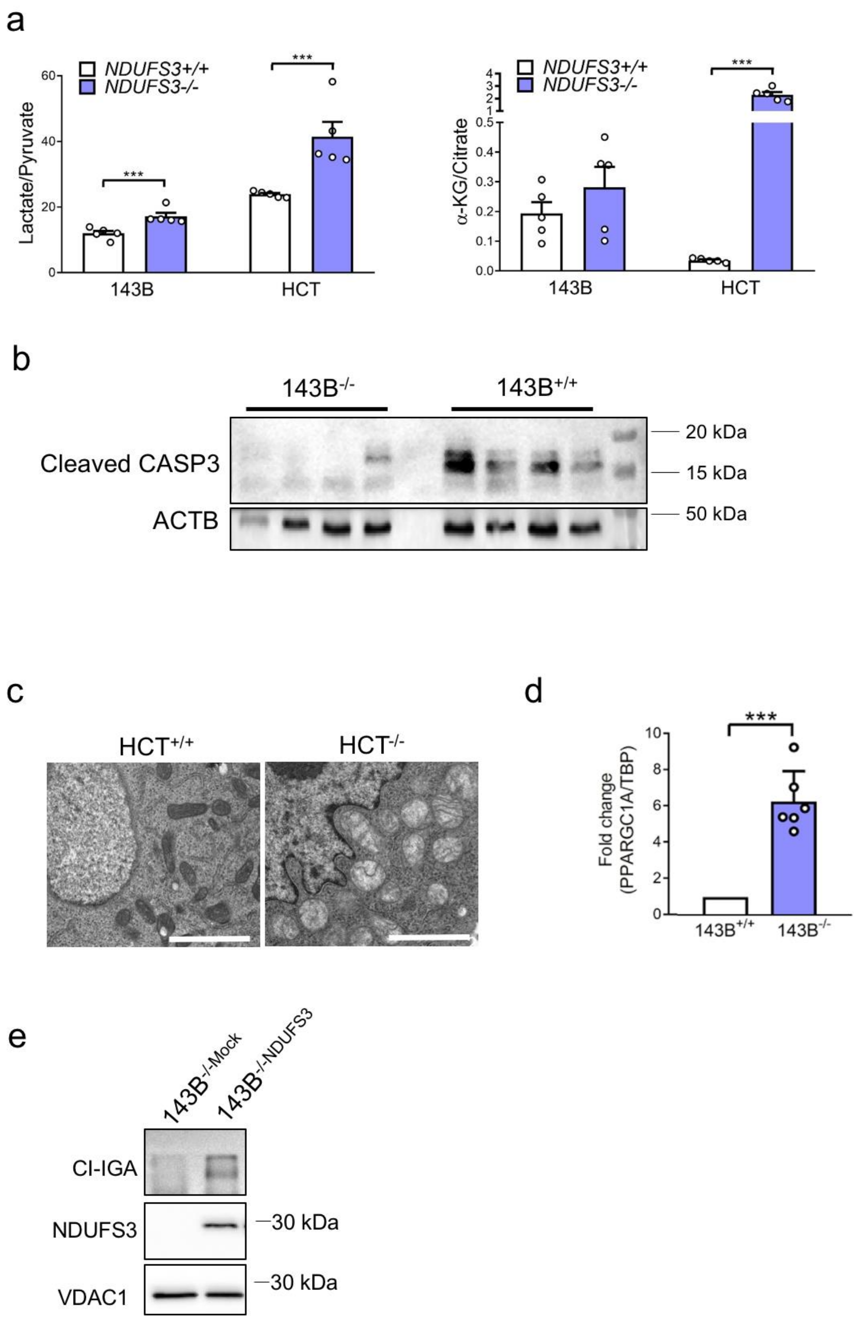

Supplementary Figure 3. Recapitulation of oncocytic phenotype by NDUFS3 KO cells and xenografts. (a) Lactate/pyruvate and α-KG/citrate ratios measured in xenograft-derived 143B [(n=5, df=8, t(lactate/pyruvate)=3.922, t(aKG/citrate)=1.135] and HCT [(n=5, df=8), t(lactate/pyruvate)=3.922, t(aKG/citrate)=10.08] cells. Data are mean+s.e.m. **(b)** Cleaved Caspase 3 western blot analysis in 143B xenografts. β-actin was used as loading control. **(c)** Evaluation of mitochondrial ultrastructure in HCT cell line. Representative electron micrographs are shown. Scale bars: 2 µm. **(d)** *PPARGC1A* expression levels in 143B xenografts evaluated by qRT-PCR. Data are mean+s.e.m. (n=6, df=10, t=9.6). Statistical significance is specified with asterisks (***$p < 0.001$). **(e)** Representative experiment of CI In-Gel Activity (CI-IGA) and western blot for NDUFS3 in $143B^{-/-}$ cells transduced with the empty vector ($143B^{-/-Mock}$) or wild-type *NDUFS3* ($143B^{-/-NDUFS3}$). VDAC1 was used as loading control.

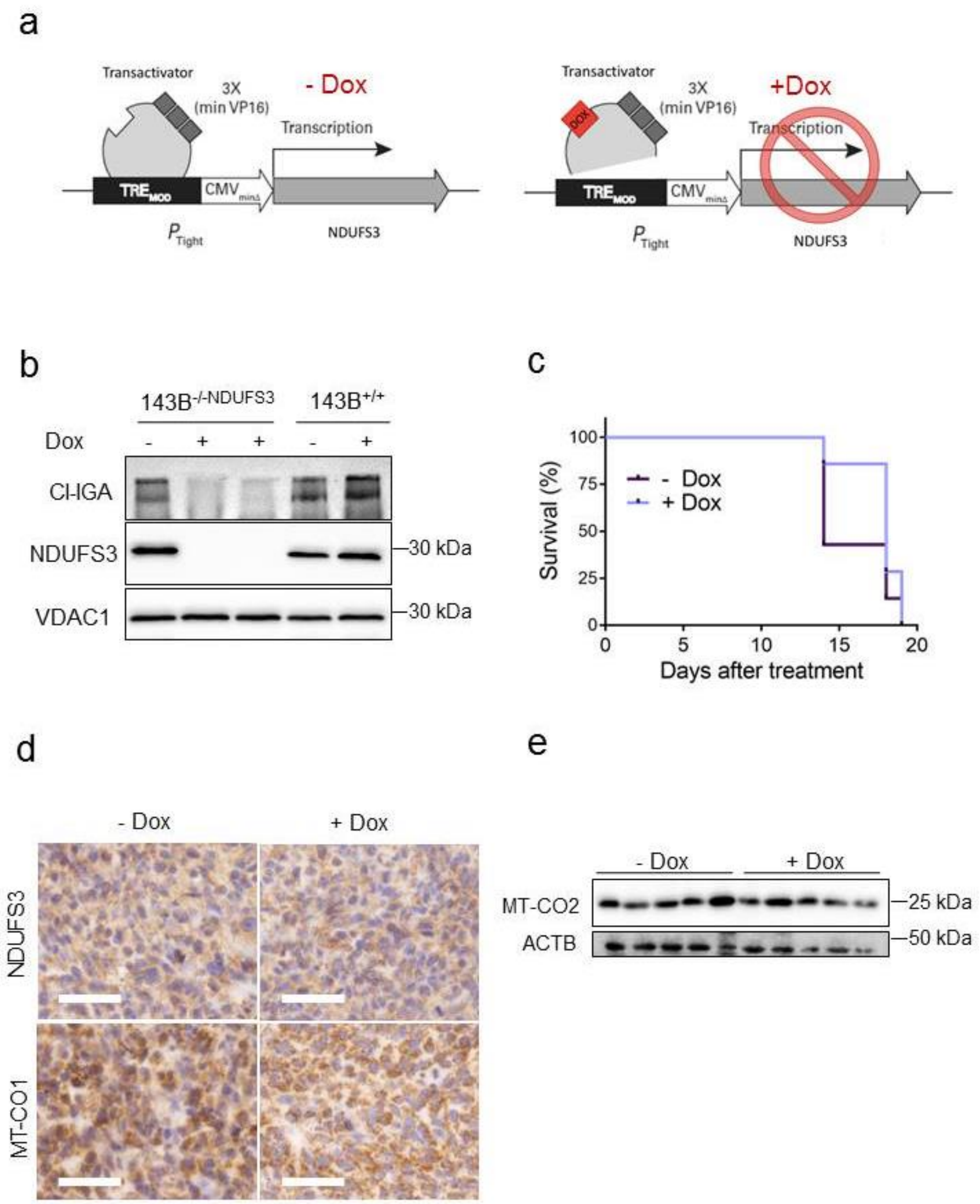

Supplementary Figure 4. Doxycyline (Dox)-inducible Tet-Off system for *NDUFS3*. (a) Scheme of Doxycyline (Dox)-inducible system which allows constitutive *NDUFS3* expression when the transactivator is active, and triggers *NDUFS3* knock-out in the presence of Dox. **(b)** Representative CI In-Gel Activity (CI-IGA) and NDUFS3 western blot in 143B cells cultured with or without Dox (100 ng mL^{-1}, 9 days). VDAC1 was used as loading control. **(c)** Kaplan-Meier curve of CD-1 nude mice injected with CI-competent 143B cells and treated with or without Dox (1 mg mL^{-1} in drinking water). Survival end-point: xenografts reaching 10% of animal weight. **(d)** Representative images of immunohistochemical staining for nuclear- (NDUFS3) and mitochondrial DNA-encoded (MT-CO1) subunits of OXPHOS complexes in $143B^{+/+}$ xenografts derived from CD-1 nude mice treated with or without Dox (1 mg mL^{-1} in drinking water). Scale bars: 50 µm. **(e)** MT-CO2 western blot analysis in $143B^{+/+}$ xenografts derived from CD-1 nude mice treated with or without Dox (1 mg mL^{-1} in drinking water). β-actin was used as loading control.

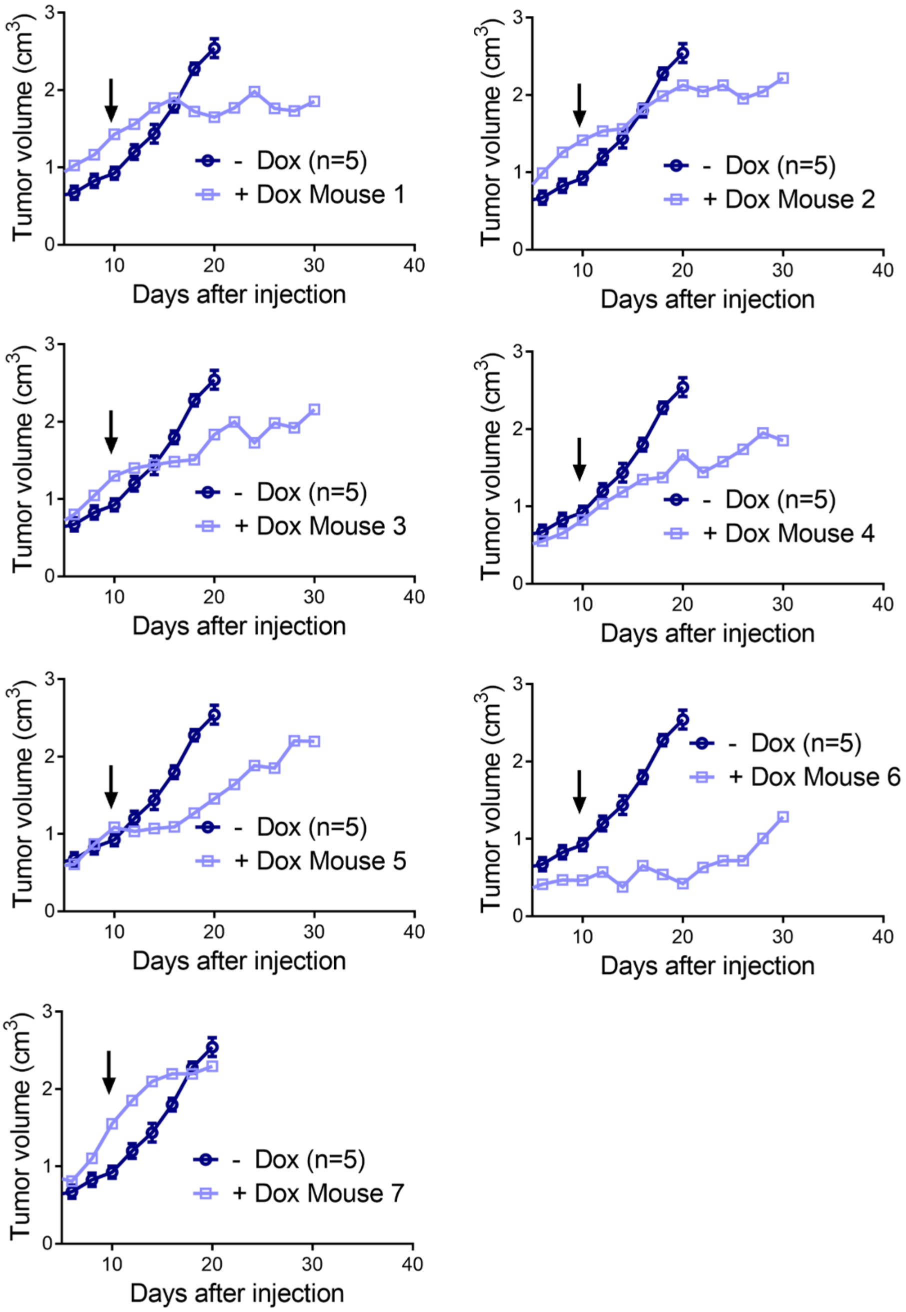

Supplementary Figure 5. Growth curves of xenografts developed by individual animals in which CI knockout was induced by Dox treatment. CD-1 nude mice were injected with 143B$^{-/-}_{NDUFS3}$ cells and treated with Dox (1 mg mL^{-1} in drinking water) when reaching 500 mm^3 (light blue). As control, the tumor growth curve presenting the average values relative to the untreated group (dark blue, n=5, data are mean±s.e.m.) is shown in each graph. The arrows indicate the timing at which inducible *NDUFS3* ablation induces CI deficiency (9 days).

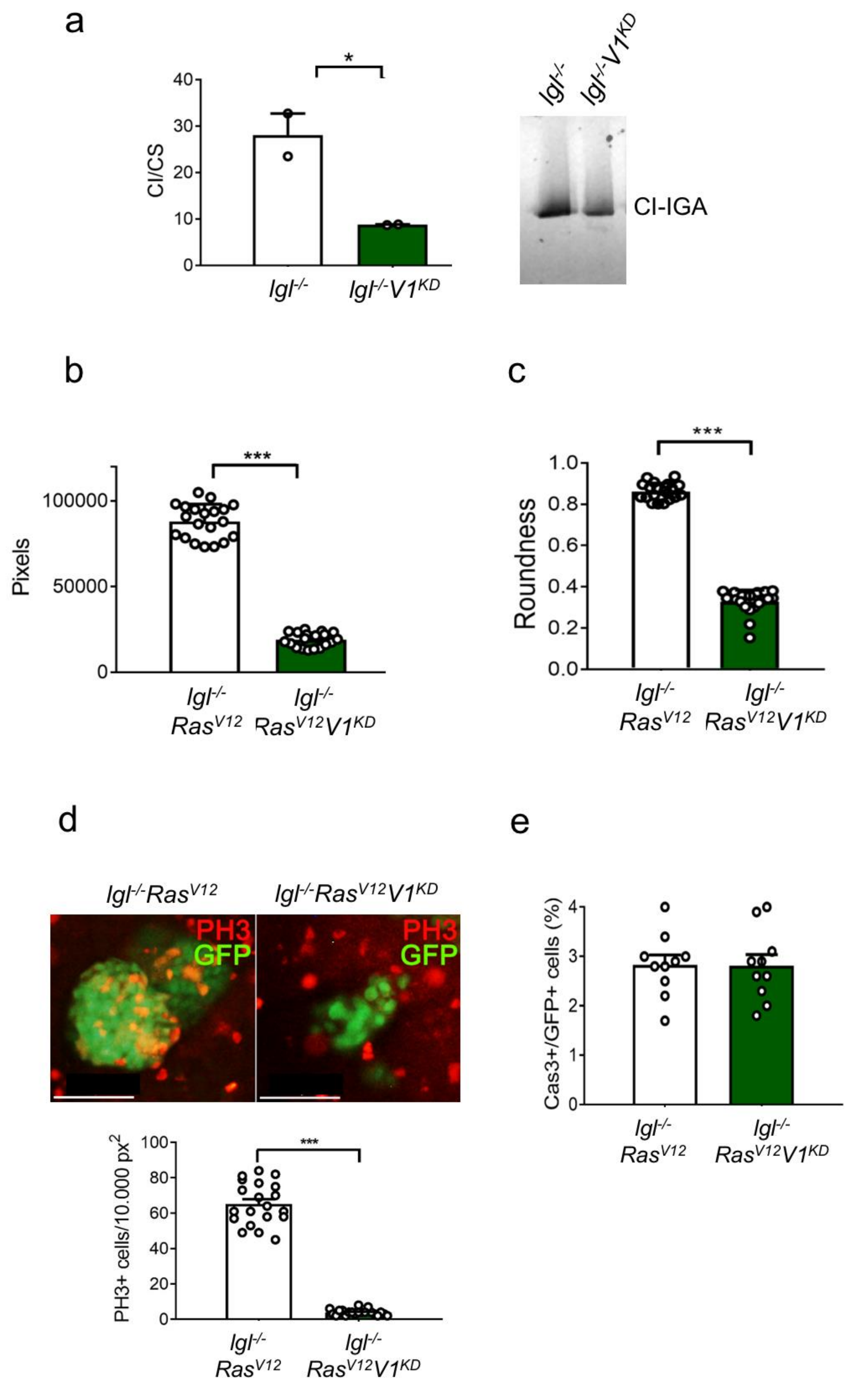

Supplementary Figure 6. CI impairment impinges on tumor proliferation in *Drosophila melanogaster* cancer models. (a) CI spectrophotometric activity normalized on citrate synthase activity (CI/CS) and CI In-Gel Activity (CI-IGA) in CI-competent (*lgl*$^{-/-}$) and deficient (*lgl*$^{-/-}$*V1*^{KD}) epithelial tumors of the fly (data were log transformed, n=2, df=2, t=6.9). Data are mean+s.e.m. **(b)** Average tumor size (GFP^{+} pixels, n=20, df=24, t=28.5) and **(c)** corresponding roundness coefficient (n=20, df=38, t=34.6) of tumorous *Drosophila* wing discs was determined in control (*lgl*$^{-/-}$*Ras*^{V12}) and CI-deficient (*lgl*$^{-/-}$*Ras*^{V12}*V1*^{KD}) clones. Student's t-test assuming unequal variance was applied for comparing average tumor size. Data are mean+s.e.m. **(d)** Expression of mitotic index marker PH3 in CI-competent (*lgl*$^{-/-}$) and deficient (*lgl*$^{-/-}$*V1*^{KD}) epithelial tumors of the fly. Data are mean+s.e.m. Representative images of the immunofluorescent staining are shown. Scale bars: 40 µm. **(e)** Amount of caspase 3 (Cas3) positive cells normalized on tumors cells (GFP^{+}) in CI-competent (*lgl*$^{-/-}$) and deficient (*lgl*$^{-/-}$*V1*^{KD}) *Drosophila* tumors. Data are mean+s.e.m. (n=10). Statistical significance is specified with asterisks (*p < 0.05, ***p < 0.001).

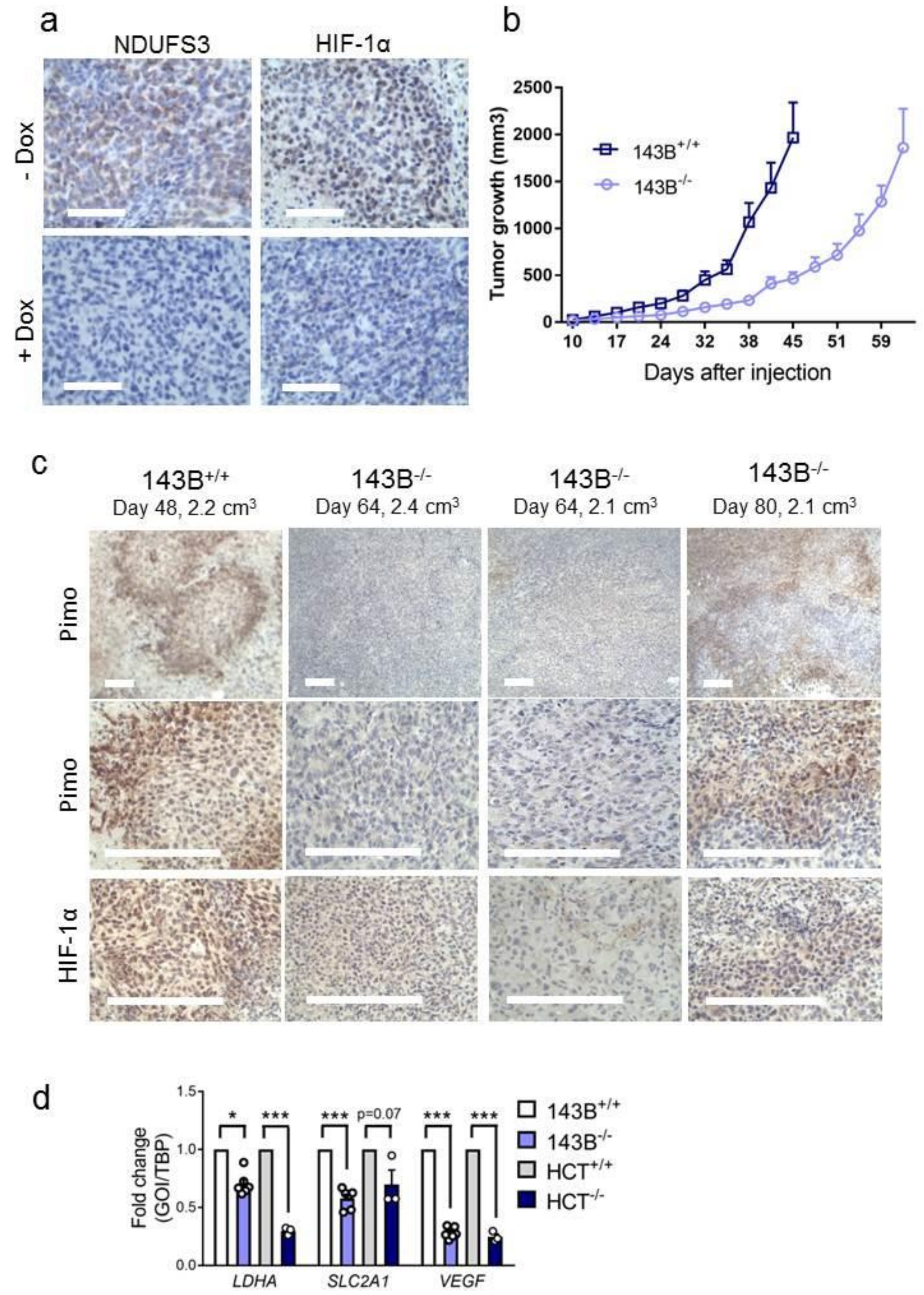

Supplementary Figure 7. HIF-1α is destabilized in NDUFS3 KO xenografts. (a) Representative immunohistochemical staining for NDUFS3 and HIF-1α in 143B$^{-/-NDUFS3}$ xenografts treated with or without Dox (1 mg mL^{-1} in drinking water). Scale bars: 50 µm. **(b)** Growth curves of 143B xenografts (n=15) in CD-1 nude mice. Data are mean±s.e.m. **(c)** Representative image of immunohistochemistry staining for hypoxia marker pimonidazole (Pimo) and HIF-1α of xenografts from CD-1 nude mice injected with 143B cells (n=12). Survival end-point: xenografts reaching 10% of animal weight. The size and the days post injection at the end-point are indicated for each tumor. Scale bars: 100 µm. **(d)** Expression levels of HIF-1α target genes evaluated by qRT-PCR in 143B [n=6, df=10, t(*LDHA*)=2.5, t(*SLC2A1*)=4.5, t(*VEGF*)=14.8] and HCT [n=3, df=4, t(*LDHA*)=11.4, t(*SLC2A1*)=2.1, t(*VEGF*)=11.3)] cells cultured in 1% O_2 for 24 hours. Data are mean+s.e.m. Statistical significance is specified with asterisks (*p < 0.05, ***p < 0.001).

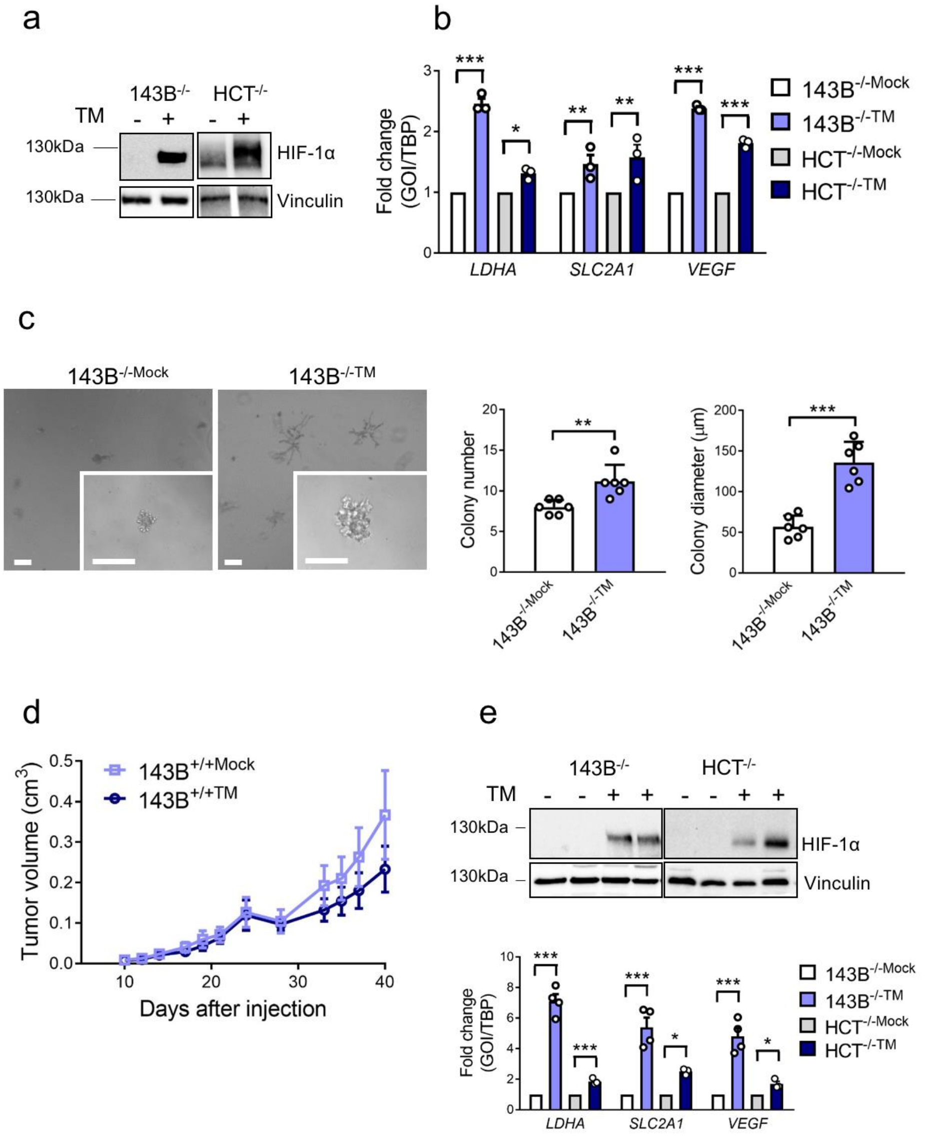

Supplementary Figure 8. Characterization of CI-deficient cells and xenografts complemented with TM-HIF-1α (TM). (a) HIF-1α western blot in CI-deficient cells expressing TM-HIF-1α (TM) or carrying the empty vector (-) cultured *in vitro* in basal conditions. Vinculin was used as loading control. **(b)** qRT-PCR of HIF-1α target genes in CI-deficient 143B (n=4, df=6, t(*LDHA*)=6.9, t(*SLC2A1*)=5.4, t(*VEGF*)=27.3) and HCT (n=3, df=4, t(*LDHA*)=38.5, t(*SLC2A1*)=5.4, t(*VEGF*)=16) cells carrying empty vector (Mock) or TM-HIF-1α (TM) cultured *in vitro* in basal conditions. Data are mean+s.e.m. (n=3). **(c)** Colony formation assay of 143B cells cultured in 3D matrigel/collagen 2:1 media. Colony number (n=6, df=10, t=3.5) and size (n=6, df=10, t=6.7) were compared. Data are mean+s.e.m. Scale bars: 200 µm. **(d)** Growth curves in CD-1 nude mice of $143B^{+/+}$ xenografts (n=6) carrying empty vector (Mock) or TM-HIF-1α (TM). Data are mean±s.e.m. **(e)** HIF-1α stabilization (western blot) and transcriptional activity (qRT-PCR) in xenografts expressing TM-HIF-1α (TM) or carrying the empty vector (-). Vinculin was used as loading control. qRT-PCR of HIF-1α target genes in CI-deficient 143B (n=4, df=6, t(*LDHA*)=13.2, t(*SLC2A1*)=8, t(*VEGF*)=6.8) and HCT (n=3, df=4, t(*LDHA*)=6.7, t(*SLC2A1*)=4.1, t(*VEGF*)=4.8) xenografts carrying empty vector (Mock) or TM-HIF-1α (TM). Data are mean+s.e.m. Statistical significance is specified with asterisks (*$p < 0.05$, **$p < 0.01$, ***$p < 0.001$).

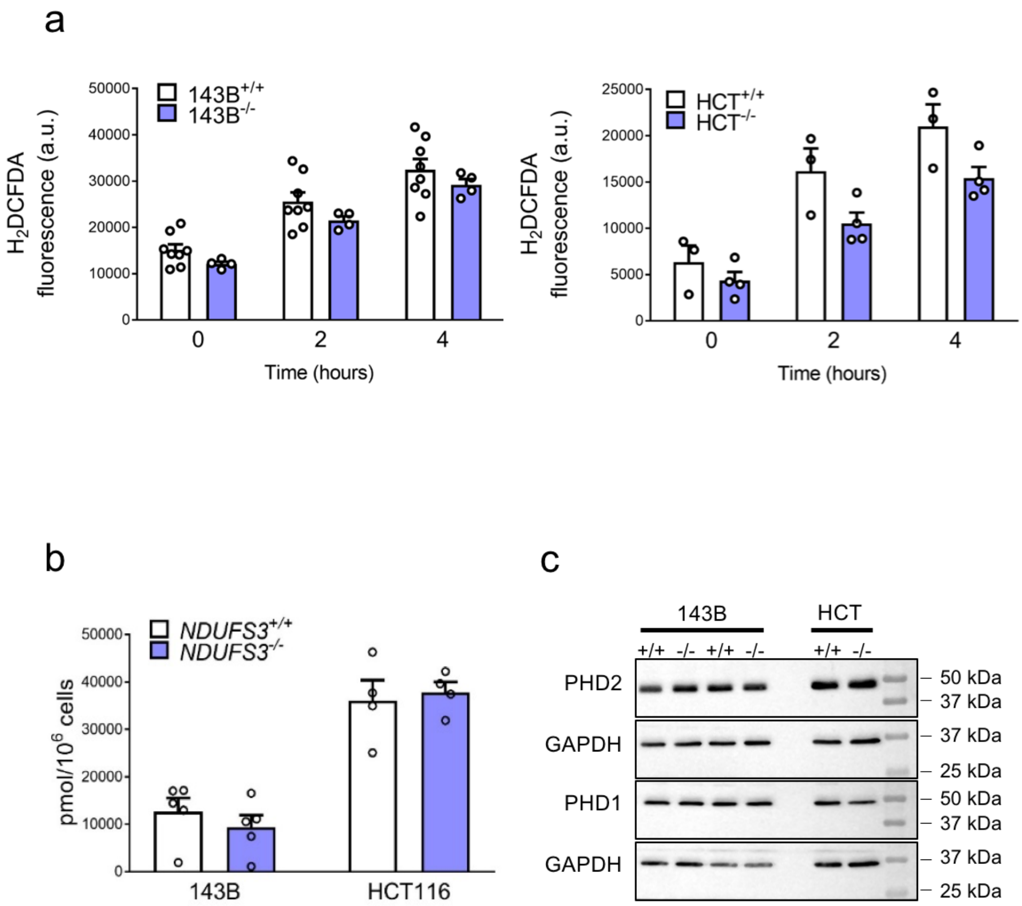

Supplementary Figure 9. HIF-1α status does not depend on oxidative stress or prolyl hydroxylases (PHDs) levels. (a) H_2O_2 production in 143B and HCT cells, determined by using H_2DCFDA probe. Data are mean+s.e.m. (n≥3). **(b)** Total glutathione (GSH+GSSG) levels in 143B and HCT cells. Data are mean+s.e.m. (n≥4). **(c)** PHD1 and PHD2 western blot analysis in 143B and HCT cells. GAPDH was used as loading control.

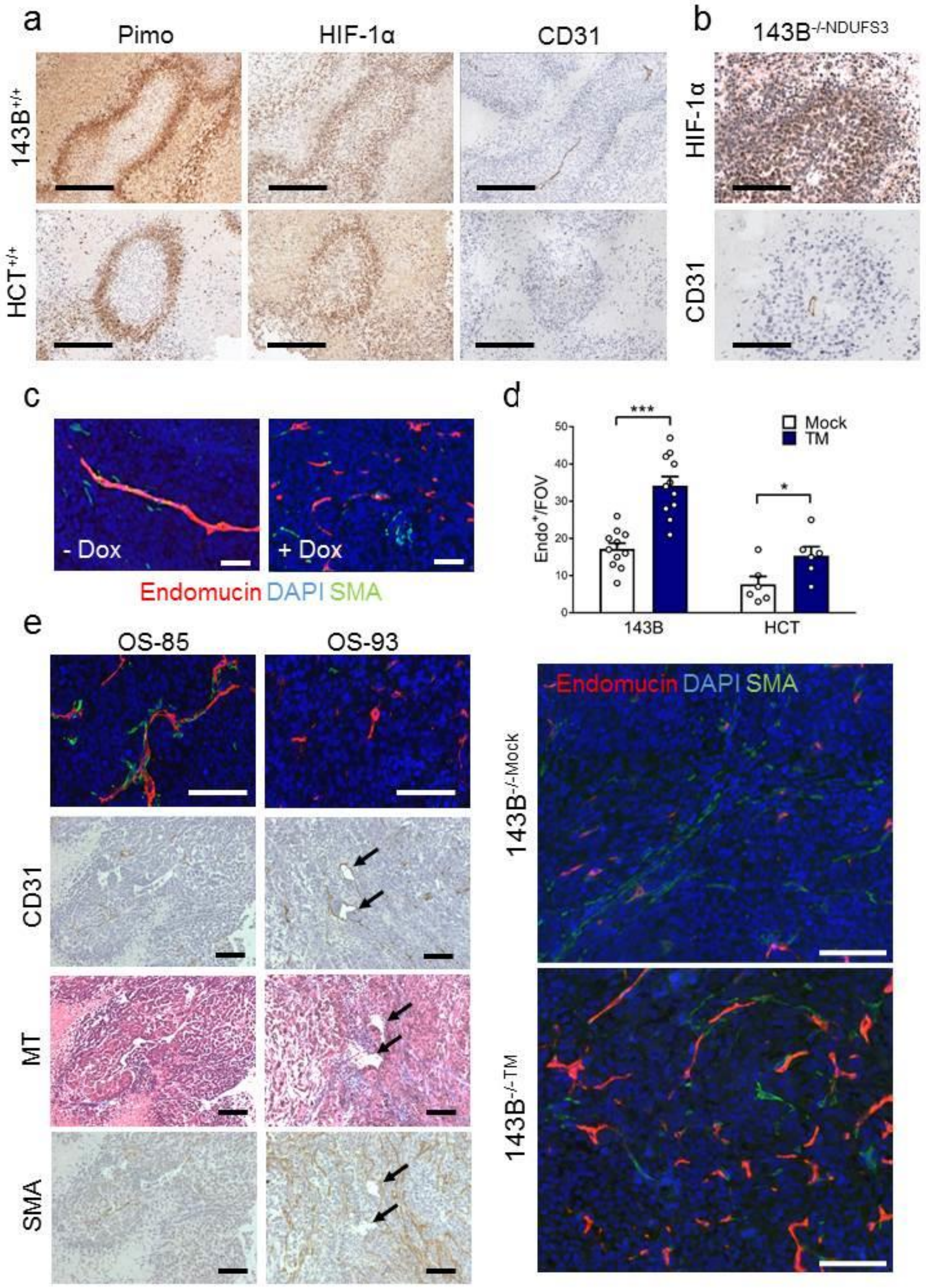

Supplementary Figure 10. Analysis of tumor angiogenesis in CI-competent and deficient tumors. (a) Representative images of immunohistochemical staining for hypoxia marker pimonidazole (Pimo), HIF-1α, and the endothelial marker CD31 in CI-competent xenografts. Scale bars: 100 µm. **(b)** Representative images of immunohistochemical analysis for HIF-1α and CD31 in $143B^{-/-NDUFS3}$ xenografts. Scale bars: 100 µm. **(c)** Representative images of immunofluorescent staining analyzing vessel morphology in $143B^{-/-NDUFS3}$ xenografts treated with or without Dox (1 mg mL^{-1} in drinking water). Scale bars: 50 µm. **(d)** The number of Endo+ structures counted in xenografts derived from cells carrying empty vector (Mock) or TM-HIF-1α (TM), in 143B (n=11, df=20, t=5.855) and HCT (n=6, df=10, t=2.375) xenografts. Representative images of the immunofluorescent staining of 143B xenografts are shown. Scale bars: 100 µm. **(e)** Representative images of immunofluorescent staining analyzing vessel morphology in xenografts derived from CI-competent (OS-85) and CI-deficient (OS-93) cells carrying the m.3571insC/*MT-ND1* mutation. Scale bars: 100 µm. Masson's trichrome (MT) and CD31 immunohistochemistry of OS-85 and OS-93 xenografts. Representative images are shown. The arrows indicate lumen-bearing vessels in CI-deficient tumors. Statistical significance is specified with asterisks (*$p < 0.05$, ***$p < 0.001$).

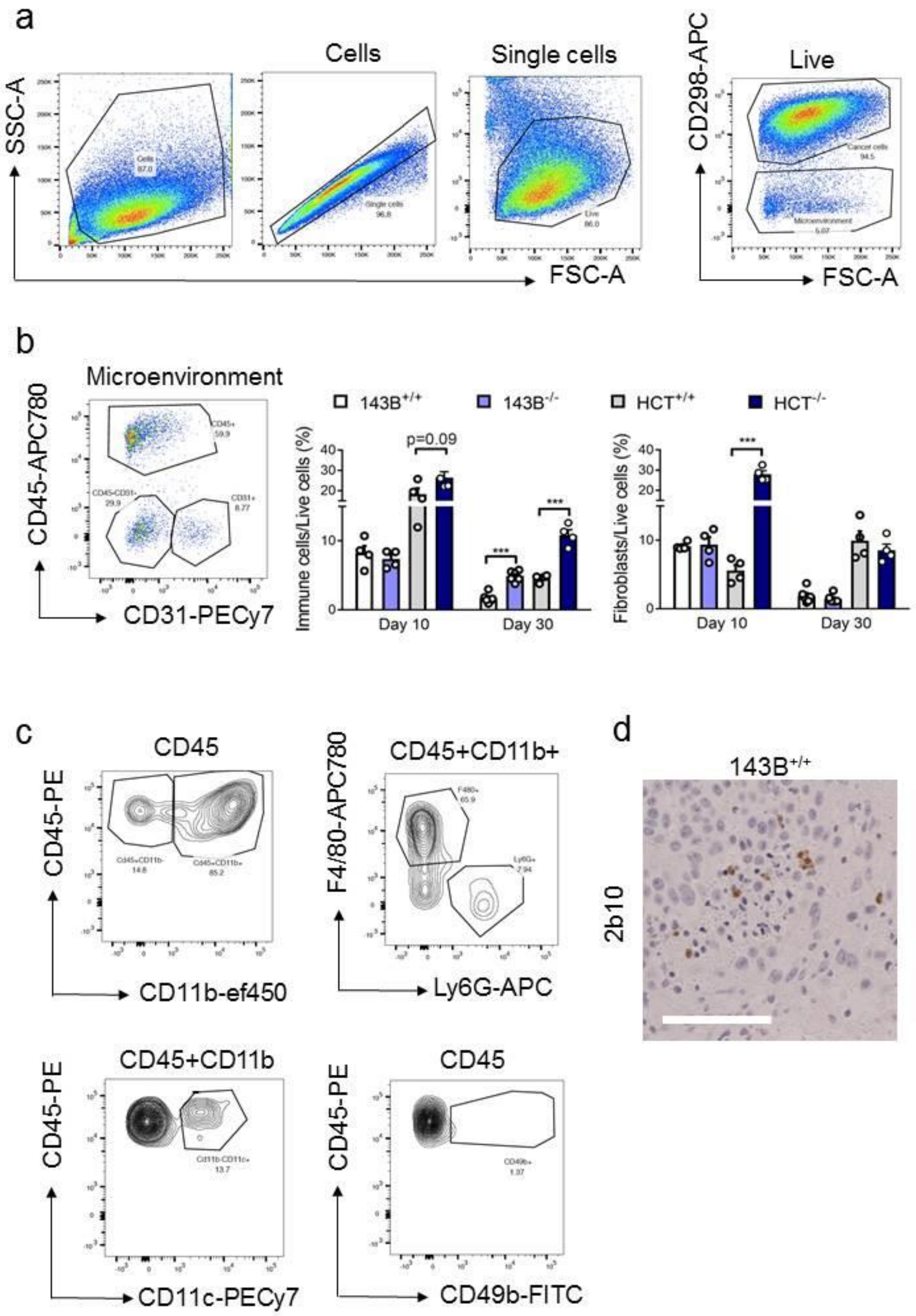

Supplementary Figure 11. Analysis of the tumor microenvironment populations in CI-deficient and control xenografts. (a) Gating strategy for characterization of tumor microenvironment (negative for human-specific marker CD298). The gates indicate population percentages. **(b)** Gating strategy and flow cytometry analysis of immune cells ($CD45^{+}CD31^{-}$) and fibroblasts ($CD45^{-}CD31^{-}$) in 143B and HCT tumors at day 10 and day 30 post-injection in ICRF nude mice. The gates indicate population percentages. Statistics data for immune cell analysis: 143B at 10 days: n=4, df=6, t=0.7; HCT at 10 days (data were log transformed) n=4, df=6, t=2; 143B at 30 days: n=6, df=10, t=7.6; HCT at 30 days: n=4, df=6, t=7.6. Statistics data for fibroblast analysis: 143B at 10 days: n=4, df=6, t=0.2; HCT at 10 days: n=4, df=6, t=11.55; 143B at 30 days: n=6, df=10, t=0.9; HCT at 30 days: n=4, df=6, t=0.8. Data are mean+s.e.m. Statistical significance is specified with asterisks (***$p < 0.001$). **(c)** Gating strategy for the analysis of the populations of the innate immune system: macrophages (CD11b+F4/80+Ly6G-), neutrophils (CD11b+F4/80-Ly6G+), dendritic cells (CD11b-CD11c+) and natural killer cells (CD45+CD49b+). The gates indicate population percentages. **(d)** Immunohistochemistry for neutrophil marker 2b10 in $143B^{+/+}$ xenografts. Scale bar: 100 µm.

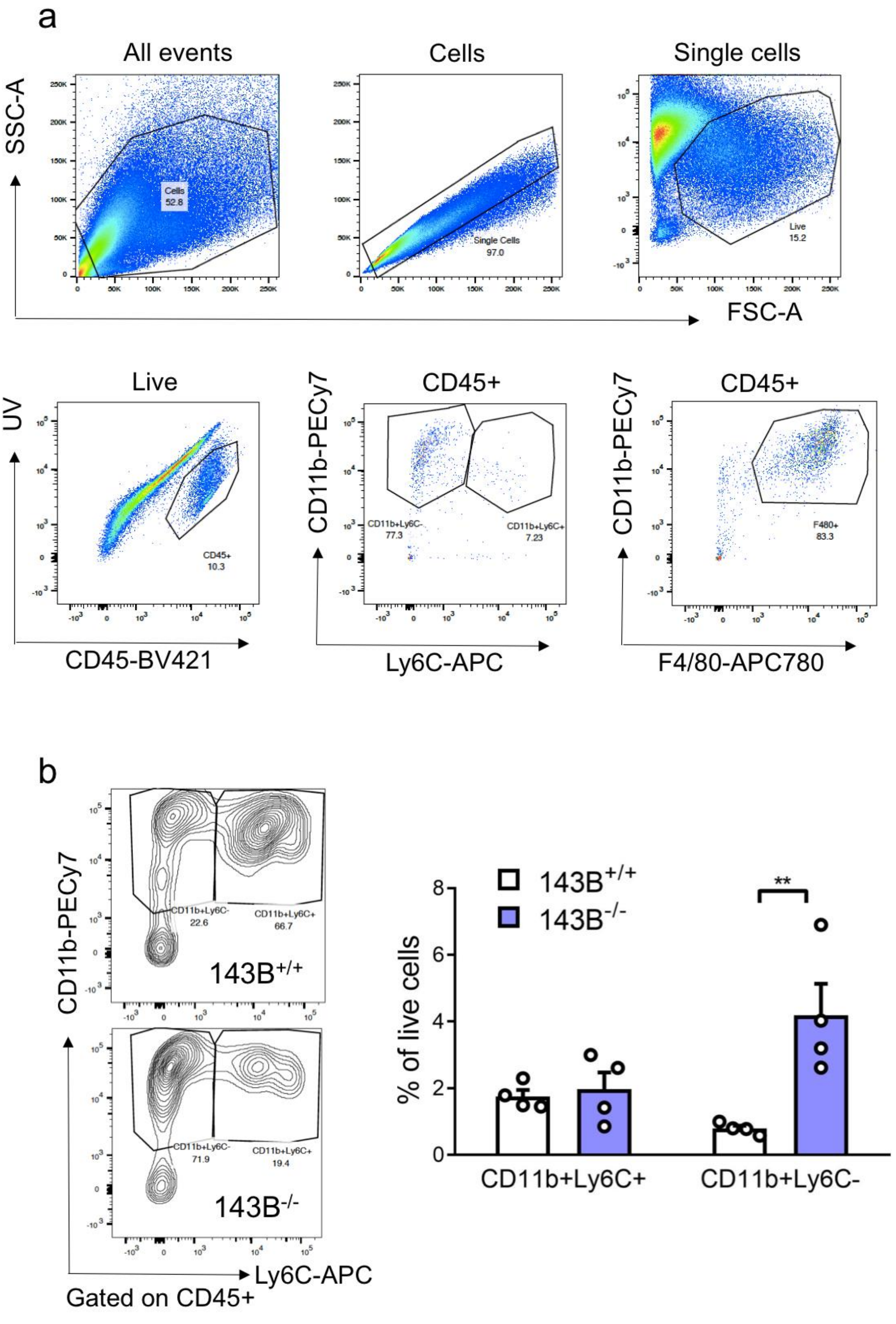

Supplementary Figure 12. Analysis of CD11b and Ly6C markers in CI-competent and deficient tumors. (a) Gating strategy for discrimination of undifferentiated (CD11b+Ly6C+) and differentiated (CD11b+Ly6C-) monocytes and macrophages (F4/80). The gates indicate population percentages. **(b)** Flow cytometry analysis of undifferentiated (CD11b+Ly6C+) and differentiated (CD11b+Ly6C-) monocytes in $143B^{+/+}$ and $143B^{-/-}$ tumors. The gates indicate population percentages. Statistical significance is specified with asterisks (**p < 0.01).

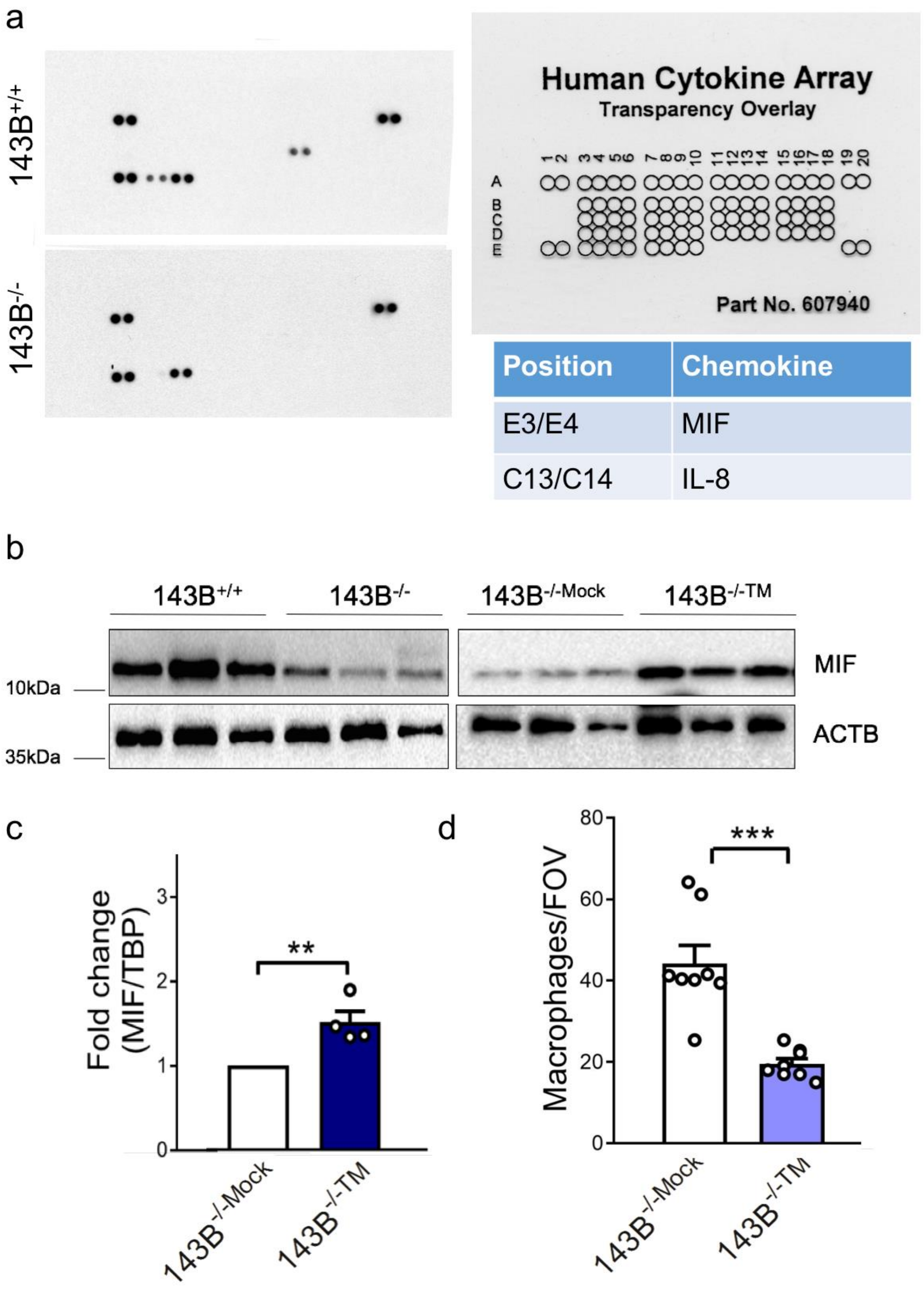

Supplementary Figure 13. Macrophage migration inhibitory factor (MIF) is downregulated in CI-deficient tumors. (a) Blotting of supernatants from 143B xenograft-derived cancer cells against the human cytokine array. Differentially detected cytokines are listed in the table. **(b)** MIF western blot analysis in xenografts derived from 143B$^{+/+}$, 143B$^{-/-}$, and 143B$^{-/-}$ cells complemented with empty vector (Mock) or TM-HIF-1α (TM). ACTB was used as loading control. **(c)** *MIF* expression evaluated by qRT-PCR in xenografts deriving from 143B$^{-/-}$ cells complemented with empty vector (Mock) or TM-HIF-1α (TM) (n=4, df=6, t=14.6). Data are mean+s.e.m. **(d)** The number of macrophages (F4/80^{+}) infiltrating tumor tissue counted per Field Of View (FOV) in xenografts (n=16, df=14, t=5.325). Data are mean+s.e.m. Statistical significance is specified with asterisks (**$p < 0.01$, ***$p < 0.001$).

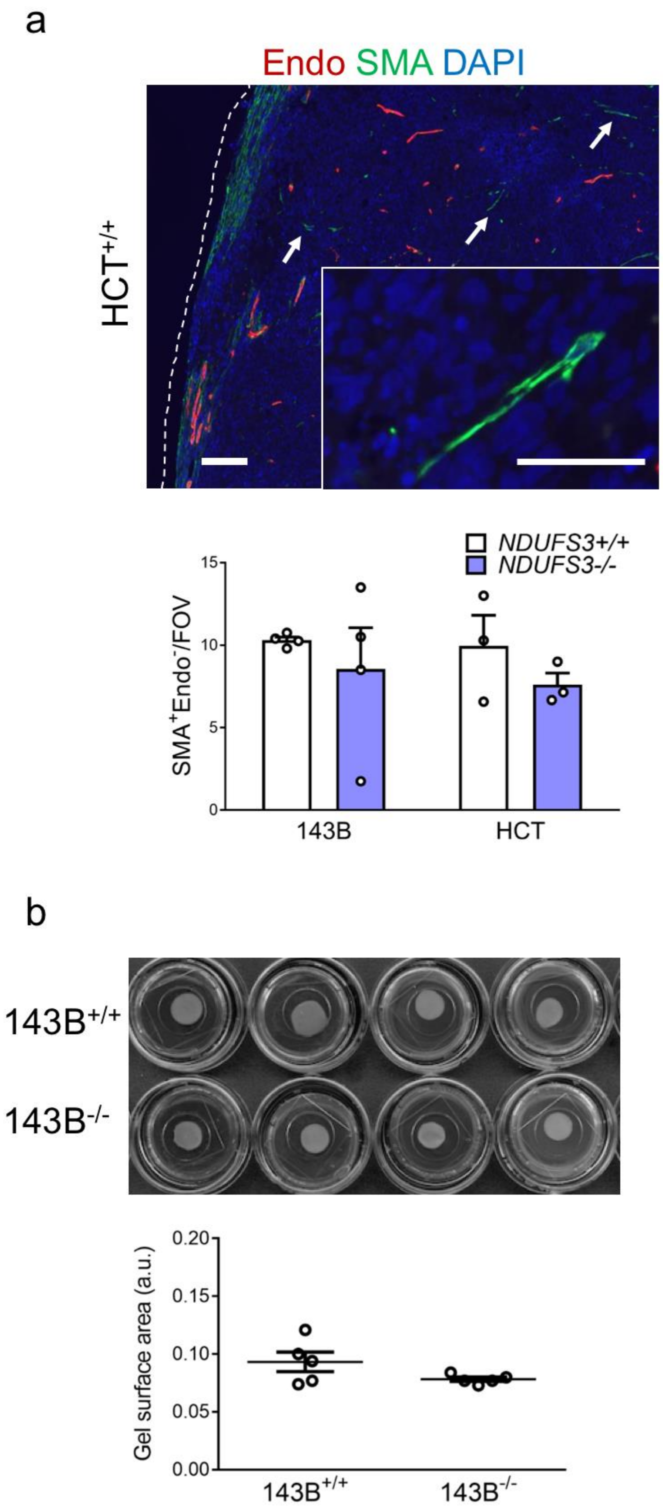

Supplementary Figure 14. Cancer associated fibroblasts in CI-deficient and control xenografts. (a) The number of single CAFs ($SMA^{+}Endo^{-}$) infiltrating tumor tissue counted per Field Of View (FOV) in 143B (n=4, df=3, t=0.7) and HCT (n=3, df=4, t=1.2) xenografts at day 30 post-injection. For $SMA^{+}Endo^{-}$ count in 143B xenografts, the Student's t-test assuming unequal variance was applied. Data are mean+s.e.m. A representative image of immunofluorescent staining is shown. The arrows indicate the single CAFs infiltrating tumor tissue counted in 143B and HCT xenografts. Dashed lines delineate the tumor margin. Scale bars: 50 µm. **(b)** *In vitro* fibroblast activation assay with conditioned media derived from 143B cells. A representative image of gel contraction at day 4 is shown. Scatter plot data are mean±s.e.m.

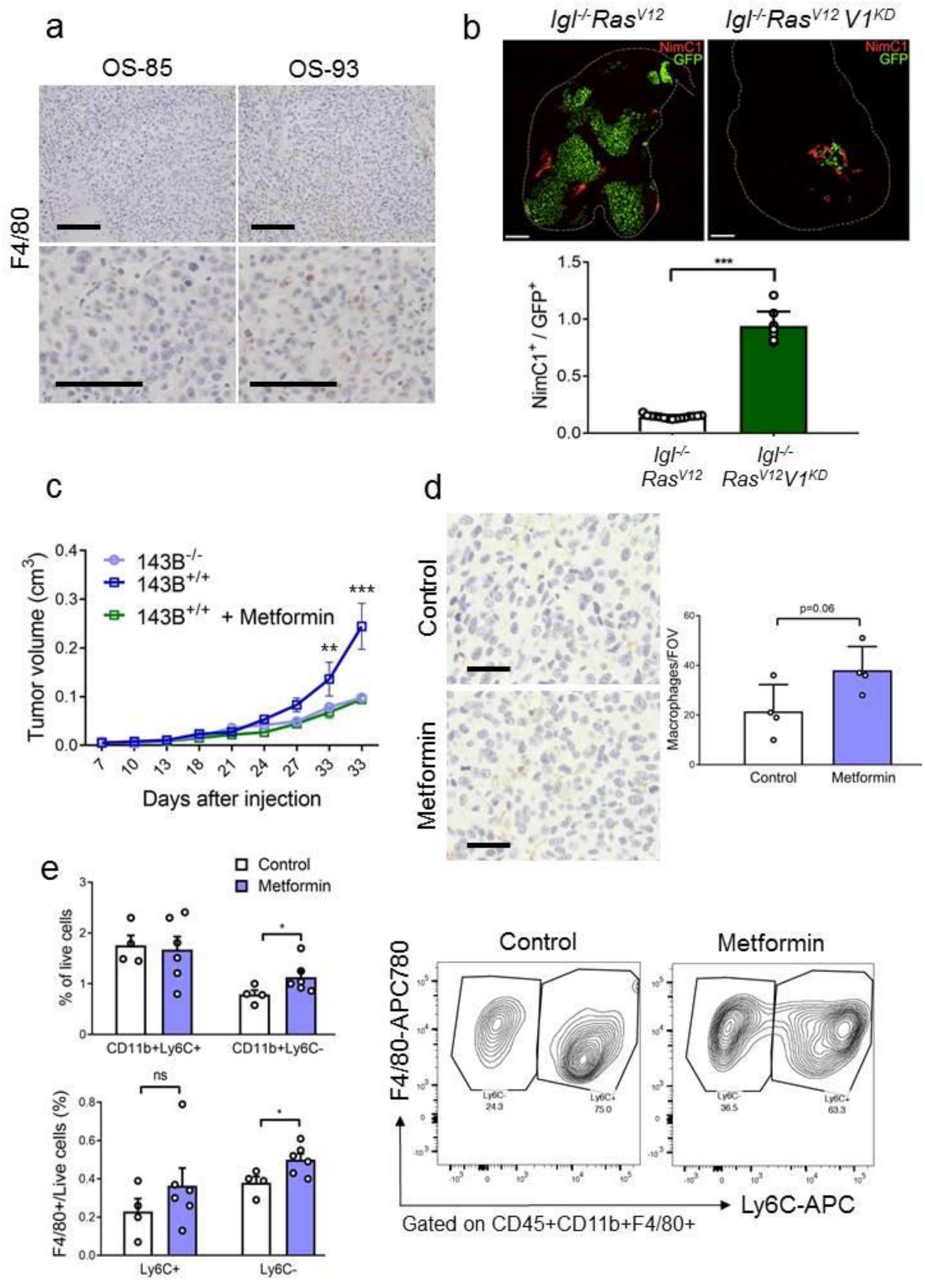

Supplementary Figure 15. Abundance of macrophages infiltrating the tumor tissue is a general property of CI-deficient tumors. (a) Representative images of immunohistochemical staining for macrophage marker F4/80 in CI-competent (OS-85) and CI-deficient (OS-93) xenografts carrying the m.3571insC/*MT-ND1* mutation. Scale bars: 100 µm. **(b)** Representative image of staining of *Drosophila* macrophages (NimC1$^+$) in control (*Ras*^{V12}) and CI-deficient (*Ras*^{V12}*V1*^{KD}) clones (n≥9, df=22, t=39.1). Dashed lines delineate the wing disc. Scale bars: 40 µm. NimC1$^+$/GFP$^+$ area per wing disc for control (*Ras*^{V12}) and CI-deficient (*Ras*^{V12}*V1*^{KD}) clones is shown. Data are mean+s.e.m. **(c)** Tumor growth curves [n=4-6, df=8, t(143B$^{+/+}$ versus Metformin, day33)=3.938] of 143B$^{-/-}$ xenografts in ICRF nude mice, treated with metformin (2 mg mL^{-1} in drinking water). Data are mean±s.e.m. **(d)** The number of macrophages (F4/80$^+$) infiltrating tumor tissue counted per Field Of View (FOV) in control and metformin-treated xenografts (n=4, df=6, t=2.290). A representative image of immunohistochemistry analysis for macrophage marker F4/80$^+$ in xenografts is shown. Scale bars: 50 µm. Data are mean+s.e.m. **(e)** Flow cytometry analysis of undifferentiated (CD11b+Ly6C+) and differentiated (CD11b+Ly6C-) monocytes and macrophages (F4/80) in control (n=4) and metformin-treated (n=6) 143B$^{-/-}$ xenografts. The gates indicate population percentages. Upper panel df=8, t=2.190. Lower panel df=8, t=2.910 Data are mean+s.e.m. Statistical significance is specified with asterisks (*p < 0.05, **p < 0.01, ***p < 0.001).

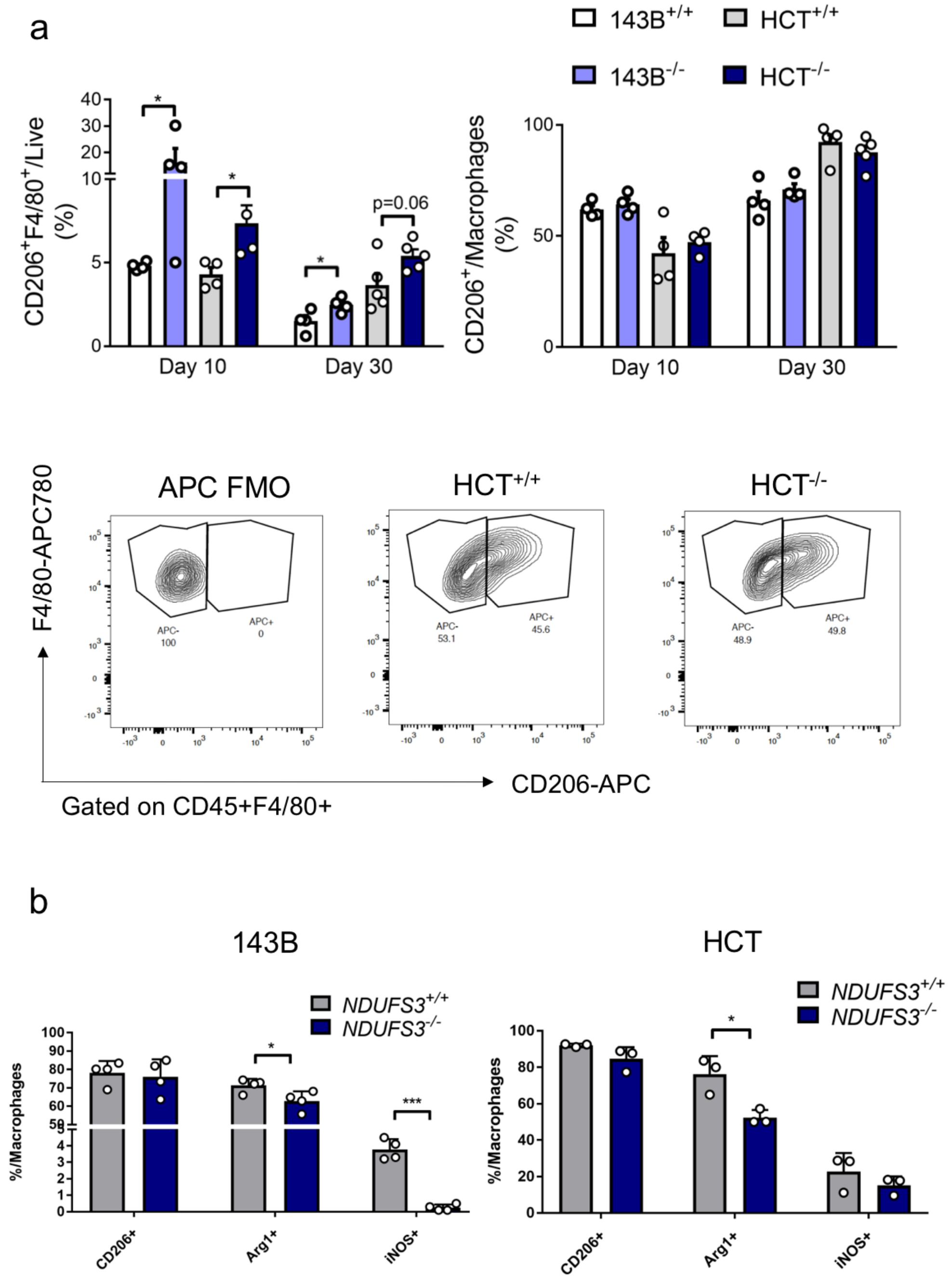

Supplementary Figure 16. CI-deficient tumors do not display increased potential to promote M1 to M2 macrophage polarization. (a) Flow cytometry analysis of $CD206^+$ macrophages in 143B and HCT tumors at day 10 and 30 post-injection in ICRF nude mice. The gates indicate population percentages. Statistics data for CD206/Macrophages: 143B at 10 days: n=4, df=6, t=0.8; HCT at 10 days: n=4, df=6, t=0.7; 143B at 30 days: n=4, df=6, t=1.1; HCT at 30 days: n=5, df=8, t=1. Statistics data for CD206/Live: 143B at 10 days: n=4, df=6, t=2.8; HCT at 10 days: n=4, df=6, t=2.7; 143B at 30 days: n=4, df=6, t=2.6; HCT at 30 days: n=5, df=8, t=2.4. For 143B at 10 days, the Student's t-test assuming unequal variance was applied. Data are mean+s.e.m. Representative contour plots are shown. FMO-Fluorescence Minus One. **(b)** Flow cytometry analysis of $CD206^+$, $Arg1^+$ and $iNOS^+$ macrophages in 143B and HCT tumors at day 30 post-injection in ICRF nude mice. Statistics 143B: n=4, df=6, t(Arg1)=2.64, t(iNOS)=10.72. Statistics HCT: n=3, df=4, t(Arg1)=3.849. Data are mean+s.e.m. Statistical significance is specified with asterisks (*$p < 0.05$, ***$p < 0.001$)

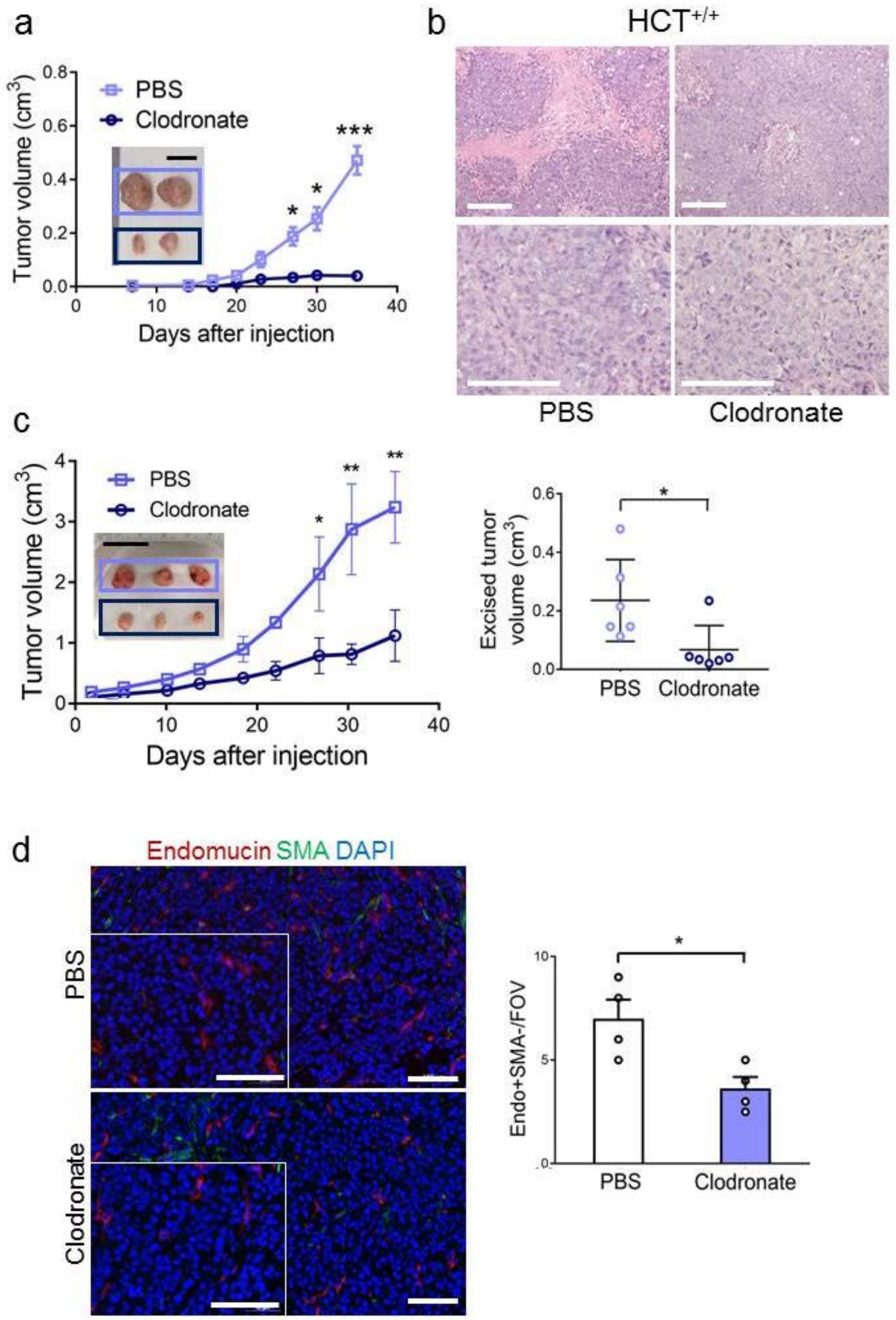

Supplementary Figure 17. The evaluation of clodronate treatment on tumor progression of CI-competent and deficient tumors. (a) Tumor growth curves [n=6, df=10, t(day 35)=5.59] of HCT$^{+/+}$ xenografts in ICRF nude mice treated with or without clodronate. Representative tumors are shown. Scale bar: 1 cm. **(b)** Haematoxylin/Eosin (HE) staining of HCT$^{+/+}$ xenografts in ICRF nude mice treated with or without clodronate. Scale bars: 50 µm. Data are mean±s.e.m. **(c)** Tumor growth curves [data were log transformed, n=6, df=10, t(day33)=3.3] and excised tumor volume (n=6, df=10, t=2.5) of 143B$^{-/-}$ xenografts in *Rag1$^{-/-}$FVB* mice treated with or without clodronate. Data are mean±s.e.m. Representative tumors are shown. Scale bar: 2 cm. **(d)** Representative images of immunofluorescent staining analyzing vessel morphology in 143B$^{-/-}$ xenografts treated with or without clodronate (n=4, df=6, t=3.16). Scale bars: 100 µm. Data are mean+s.e.m. Statistical significance is specified with asterisks (*p<0.05, **p < 0.01, ***p<0.001).

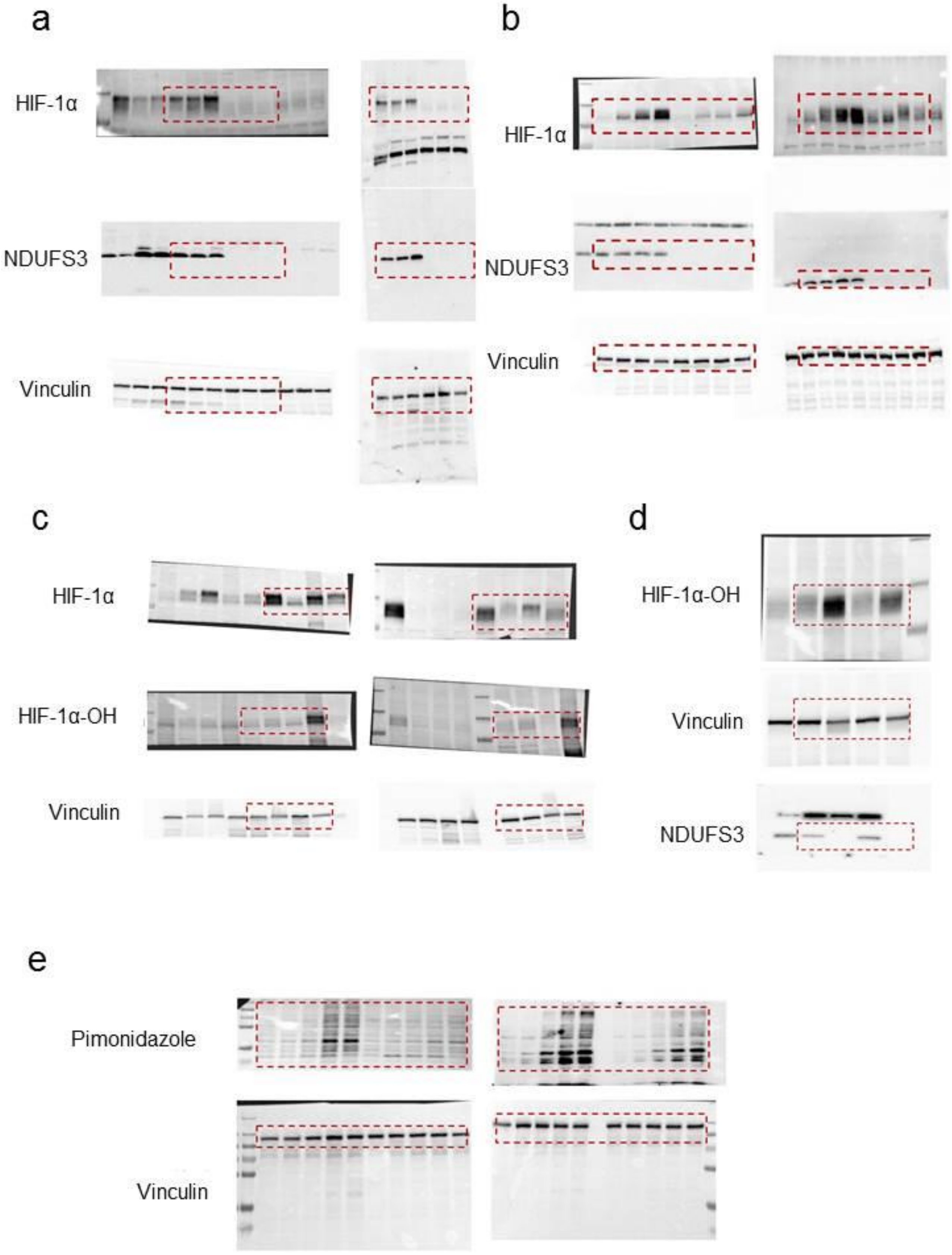

Supplementary Figure 18. The uncropped blots for the data presented in main figures. (a) Blots relative to Fig. 3a. **(b)** Blots relative to Fig. 3e. **(c)** Blots relative to Fig. 4b. **(d)** Blots relative to Fig. 4c. **(e)** Blots relative to Fig. 4e.

Supplementary Table 1. Names and sequences of primers used for qRT-PCR. Bp-base pairs

Name	ENTREZ Gene ID	Sequence	Product length (bp)	Conc. in PCR (µM)	Amplification Efficiency
SLC2A1_F	6513	ACTCCATCATGGGCAACAAG	222	0.2	98%
SLC2A1_R		TCTGCCGACTCTCTTCCTTC		0.4	
LDHA_F	3939	TGGGAGTTCACCCATTAAGC	182	0.4	96%
LDHA_R		AGCACTCTCAACCACCTGCT		0.4	
VEGFA_F	7422	ACGAGGGCCTGGAGTGTGT	58	0.2	99.7%
VEGFA_R		CGCATAATCTGCATGGTGATG		0.4	
MIF_F	4282	AGAACCGCTCCTACAGCAAG	121	0.4	97%
MIF_R		GAGTTGTTCCAGCCCACATT		0.4	
PPARGC1A _F	10891	GAGAGTCTGAGAGGGCCAAG	150	0.4	96%
PPARGC1A _R		TGCACTCCTCAATTTCACCA		0.4	
TBP_F	6908	TGCACAGGAGCCAAGAGTGAA	132	0.4	105%
TBP_R		CACATCACAGCTCCCCACCA		0.6	
