# Supplementary material for: Inducing cancer indolence by targeting mitochondrial Complex I is potentiated by blocking macrophage-mediated adaptive responses
Source: Nat Commun. 2019 Feb 22;10:903. doi: 10.1038/s41467-019-08839-1 (PMC6385215; doi:10.1038/s41467-019-08839-1)
Supplement: Supplementary file 3 — Reporting Summary [file 41467_2019_8839_MOESM3_ESM.pdf]

nature research

Corresponding author(s): Giuseppe Gasparre
Anna Maria Porcelli
Ilaria Malanchi

Reporting Summary

Nature Research wishes to improve the reproducibility of the work that we publish. This form provides structure for consistency and transparency in reporting. For further information on Nature Research policies, see Authors & Referees and the Editorial Policy Checklist.

Statistical parameters

When statistical analyses are reported, confirm that the following items are present in the relevant location (e.g. figure legend, table legend, main text, or Methods section).

n/a	Confirmed	
☐	☒	The exact sample size (*n*) for each experimental group/condition, given as a discrete number and unit of measurement
☐	☒	An indication of whether measurements were taken from distinct samples or whether the same sample was measured repeatedly
☐	☒	The statistical test(s) used AND whether they are one- or two-sided<br>*Only common tests should be described solely by name; describe more complex techniques in the Methods section.*
☐	☒	A description of all covariates tested
☐	☒	A description of any assumptions or corrections, such as tests of normality and adjustment for multiple comparisons
☐	☒	A full description of the statistics including central tendency (e.g. means) or other basic estimates (e.g. regression coefficient) AND variation (e.g. standard deviation) or associated estimates of uncertainty (e.g. confidence intervals)
☐	☒	For null hypothesis testing, the test statistic (e.g. *F*, *t*, *r*) with confidence intervals, effect sizes, degrees of freedom and *P* value noted<br>*Give P values as exact values whenever suitable.*
☒	☐	For Bayesian analysis, information on the choice of priors and Markov chain Monte Carlo settings
☒	☐	For hierarchical and complex designs, identification of the appropriate level for tests and full reporting of outcomes
☒	☐	Estimates of effect sizes (e.g. Cohen's *d*, Pearson's *r*), indicating how they were calculated
☐	☒	Clearly defined error bars<br>*State explicitly what error bars represent (e.g. SD, SE, CI)*

Our web collection on statistics for biologists may be useful.

Software and code

Policy information about availability of computer code

Data collection	Metamorph software (Universal Imaging), BD FACSDIVA Software (BD Bioscience), ZEN (Carl Zeiss Microscopy GmbH, Germany), ImageLab software version 5.1, 7500 Software v2.3 (Applied Biosystems), Run3730 Data Collection v3.0 (Applied Biosystems)
Data analysis	ImageJ (NIH), Flow Jo (Tree Star Inc) software, Microsoft Excel (Office 2010), GraphPad Prism version 7 (GraphPad Software Inc., San Diego, CA, USA)

For manuscripts utilizing custom algorithms or software that are central to the research but not yet described in published literature, software must be made available to editors/reviewers upon request. We strongly encourage code deposition in a community repository (e.g. GitHub). See the Nature Research guidelines for submitting code & software for further information.

Data

Policy information about availability of data

All manuscripts must include a data availability statement. This statement should provide the following information, where applicable:

- Accession codes, unique identifiers, or web links for publicly available datasets
- A list of figures that have associated raw data
- A description of any restrictions on data availability

All relevant data are available in the article and in the Supplementary data file. Any further detail is available from the corresponding author on reasonable request.

Field-specific reporting

Please select the best fit for your research. If you are not sure, read the appropriate sections before making your selection.

☒ Life sciences ☐ Behavioural & social sciences ☐ Ecological, evolutionary & environmental sciences

For a reference copy of the document with all sections, see nature.com/authors/policies/ReportingSummary-flat.pdf

Life sciences study design

All studies must disclose on these points even when the disclosure is negative.

Sample size	Sample size was determined based on the numbers reported in the field.
Data exclusions	Data from animals with ulcerating tumors ere excluded from the analyses. Such exclusion criteria were pre-established.
Replication	For each experiment, at least a triplicate biological replicates were analyzed. In vitro analyses were repeated at least by two independent experiments. In vivo experiments were repeated at least twice for data presented in Fig. 1a, Fig. 3g, Fig. 5a-c, Fig. 6b, Fig. 6d, Supplementary Figure 16a and Figure 17c.
Randomization	For xenograft growth studies, the animals deriving from different cages at arrival were grouped together and then randomly selected for distribution in experimental groups. When possible, different experimental group subjects were kept together in the same cage (not valid for doxycycline experiment-Fig.2a and metformin+PLX experiment Fig.7e).
Blinding	The histopathology analyses were blinded with a pathologist (MR) evaluating data produced by biologists (IK and RV).

Reporting for specific materials, systems and methods

Materials & experimental systems

n/a	Involved in the study	
☐	☒	Unique biological materials
☐	☒	Antibodies
☐	☒	Eukaryotic cell lines
☒	☐	Palaeontology
☐	☒	Animals and other organisms
☒	☐	Human research participants

Methods

n/a	Involved in the study	
☒	☐	ChIP-seq
☐	☒	Flow cytometry
☒	☐	MRI-based neuroimaging

Unique biological materials

Policy information about availability of materials

Obtaining unique materials	All unique materials used are available from the corresponding authors on request.

Antibodies

Antibodies used

Following primary antibodies were used for western blot: anti-NDUFS3 (AbCam #177471) 1:1000/1-hour at room temperature (RT); anti-HIF1α (GeneTex #GTX127309) 1:2000/1-hour at RT; anti-Vinculin (Sigma-Aldrich #V9131) 1:10000/1-hour at RT; anti-pimonidazole (Hydroxyprobe #4.3.11.3) 1:3000/2-hours at 37°C; anti-HIF-1α-OH (Cell Signaling #3434) 1:500/O/N at 4°C; anti-VDAC (AbCam #154856) 1000/2-hours at RT; anti-MT-CO2 (AbCam #110258) 1:1000/2-hours at RT; anti-MIF (AbCam #175189) 1:5000/1-hour at RT; anti-ACTB (Santa Cruz #SC-1615) 1:500/1-hour at RT and MIF (AbCam #175189); anti-cleaved Caspase 3 (Cell Signaling Technology #9661) 1:1000/O/N at 4°C; anti-PHD1 (Abcam #ab108980) 1:1000/ O/N at 4°C, anti EGLN1/PHD2 (Novus Bio #NB100-137) 1:1000/ O/N at 4°C; anti-GAPDH (Sigma Aldrich #G8795) 1:20000 2-hours at RT.

The following primary antibodies were used for IHC: rabbit monoclonal anti-NDUFS3 (1:200, Abcam #177471); rabbit polyclonal anti-HIF-1α (1:350, Sigma-Aldrich #HPA001275); mouse monoclonal anti-pimonidazole (1:400, Hypoxyprobe #Mab-4.3.11.3); rabbit monoclonal anti-CD-31 (1:50; Abcam #28364); mouse monoclonal anti-KI-67 (1:100, Dako #M7240); mouse monoclonal anti-MTCOX1 (1:1000, Abcam #14705); mouse monoclonal anti-NDUFS4 (1:1000, Abcam #55540) and rat monoclonal F4/80 (1:100, eBiosciences #14-4801). Neutrophil marker 2b10 antibody was developed in house at The Francis Crick Institute.

The following conjugated antibodies were used for flow cytometry: anti-CD298-APC (clone LNH-94, Biolegend #341706), anti-

	CD45-APC780 (clone 30-F11, eBioscience #47-0451-80), anti-CD31-PeCy7 (clone 390, eBioscience #25-0311-82); anti anti-CD45-PE (clone 30-F11, eBioscience #12-0451-82), anti-CD11b-ef450 (clone M1/70, eBioscience #48-0112-82), anti-F4/80-APC780 (clone BM8, eBioscience #47-4801-80), anti-Ly6G-APC (clone 1A8, BD Bioscience #560599), anti-CD11c-PeCy7 (clone N418, Biolegend #117317); anti-CD49b-FITC (clone 30-F11, Biolegend #108905, anti-CD45-BV421 (Biolegend #103133), anti-F4/80-APC780 (clone BM8, eBioscience #47-4801-80), anti-CD206-APC (clone C068C2, Biolegend #141707), anti-Arg1-PECy7 (clone A1exF5, eBiosceience #25-3697-82), anti-iNOS-PE (CXNPT, eBioscieence #12-5920-82), anti-CD45-BV421 (Biolegend #103133), anti-CD11b (clone M1/70, eBioscience #48-0112-82), anti-Ly6C-APC (Biolegend #128016) and anti-F4/80-APC780 (clone BM8, eBioscience #47-4801-80.
Validation	NDUFS3 antibody was validated by the successful genetic knock-out of the protein. HIF-1a antibodies were validated by analyzing lyzates deriving from cells cultured in hypoxia. Anti-CD298 human specificity was validated by running a mouse tissue only sample.

Eukaryotic cell lines

Policy information about cell lines

Cell line source(s)	143B cells were purchased from ATCC (#CRL-8303) and HCT116 cells were a kind gift from Prof. Paolo Pinton from the University of Ferrara.
Authentication	STR Analysis was performed as described in the Methods, after the genome editing (2015), as well as towards the end of the study in 2017.
Mycoplasma contamination	Cell lines were regularly screened for mycoplasma contamination as described in the Methods.
Commonly misidentified lines (See ICLAC register)	No misidentified lines were used.

Animals and other organisms

Policy information about studies involving animals; ARRIVE guidelines recommended for reporting animal research

Laboratory animals	We used 5-8 week old CD1 nude mice for all experiments except Supplementary Figure 17c where Rag-/-FVB mice were used.
Wild animals	Study did not involve wild animals.
Field-collected samples	Study did not involve samples from the field.

Flow Cytometry

Plots

Confirm that:

☒ The axis labels state the marker and fluorochrome used (e.g. CD4-FITC).

☒ The axis scales are clearly visible. Include numbers along axes only for bottom left plot of group (a 'group' is an analysis of identical markers).

☒ All plots are contour plots with outliers or pseudocolor plots.

☒ A numerical value for number of cells or percentage (with statistics) is provided.

Methodology

Sample preparation	xenograft samples (approximately 50 mm3) were digested immediately after the sacrifice for 40 minutes at 37°C with Liberase TL (Sigma #5401020001), Liberase TM (Sigma #5401135001) and DNaseI (Sigma #DN25) in HBSS and passed through a 100 µm strainer. Hypotonic lysis with Red Blood Cell Lysis Buffer (Sigma #11814389001) was performed and remaining cells were washed with MACS buffer (2 mM EDTA, 0.5% BSA in PBS), blocked using FcR Blocking Reagent (Miltenyi #130-092-575) and incubated with panels of pre-labelled antibodies.
Instrument	LSRFortessa cell analyzer (BD Biosciences)
Software	BD FACSDIVA Software (BD Bioscience) and Flow Jo (Tree Star Inc) software were used.
Cell population abundance	No cell sorting was performed. All flow cytometry data regard only sample analysis.
Gating strategy	Staining with DAPI enalbed distinguishing live from dead/necrotic cells on a FSC/SSC plot. Only live single cells were considered as a starting population. In parallel to experimental sample analysis, liver, lung and a piece of tumor tissue were digested together and stained for each single color of a panel and for all colors except one (Fluorescence Minus One-FMO). Gating for each color was determined by excluding from the gate the area positive in the FMO reading.

☒ Tick this box to confirm that a figure exemplifying the gating strategy is provided in the Supplementary Information.
